# Chronic psychosocial stress during pregnancy affects maternal behavior and neuroendocrine function and modulates hypothalamic CRH and nuclear steroid receptor expression

**DOI:** 10.1038/s41398-020-0704-2

**Published:** 2020-01-16

**Authors:** Sandra P. Zoubovsky, Sarah Hoseus, Shivani Tumukuntala, Jay O. Schulkin, Michael T. Williams, Charles V. Vorhees, Louis J. Muglia

**Affiliations:** 1 Center for the Prevention of Preterm Birth, Perinatal Institute, Cincinnati Children’s Hospital Medical Center, Cincinnati, OH USA; 2 Department of Pediatrics, University of Cincinnati College of Medicine, Cincinnati, OH USA; 3 Molecular and Developmental Biology Graduate Program, University of Cincinnati College of Medicine, Cincinnati Children’s Hospital Medical Center, Cincinnati, OH USA; 4 Division of Human Genetics, Cincinnati Children’s Hospital Medical Center, Department of Pediatrics, University of Cincinnati College of Medicine, Cincinnati, OH USA; 5 Department of Neuroscience, Georgetown University, Washington, DC USA; 6 Department of Obstetrics and Gynecology, University of Washington, Seattle, WA USA; 7 Division of Neurology, Cincinnati Children’s Hospital Medical Center, Cincinnati, OH USA

**Keywords:** Molecular neuroscience, Physiology

## Abstract

Postpartum depression (PPD) affects up to 20% of mothers and has negative consequences for both mother and child. Although exposure to psychosocial stress during pregnancy and abnormalities in the hypothalamic pituitary adrenal (HPA) axis have been linked to PPD, molecular changes in the brain that contribute to this disease remain unknown. This study utilized a novel chronic psychosocial stress paradigm during pregnancy (CGS) to investigate the effects of psychosocial stress on maternal behavior, neuroendocrine function, and gene expression changes in molecular regulators of the HPA axis in the early postpartum period. Postpartum female mice exposed to CGS display abnormalities in maternal behavior, including fragmented and erratic maternal care patterns, and the emergence of depression and anxiety-like phenotypes. Dysregulation in postpartum HPA axis function, evidenced by blunted circadian peak and elevation of stress-induced corticosterone levels, was accompanied by increased CRH mRNA expression and a reduction in CRH receptor 1 in the paraventricular nucleus of the hypothalamus (PVN). We further observed decreased PVN expression of nuclear steroid hormone receptors associated with CRH transcription, suggesting these molecular changes could underlie abnormalities in postpartum HPA axis and behavior observed. Overall, our study demonstrates that psychosocial stress during pregnancy induces changes in neuroendocrine function and maternal behavior in the early postpartum period and introduces our CGS paradigm as a viable model that can be used to further dissect the molecular defects that lead to PPD.

## Introduction

Mothers in the peripartum period are uniquely sensitive to developing psychopathologies, including postpartum depression (PPD). PPD can affect up to 20% of mothers and can have detrimental effects on the mother-infant dyad^1–3^. Although substantial evidence indicates exposure to psychosocial stress is a prominent risk factor precipitating the development of PPD^4–6^, the underlying neurobiological mechanisms that contribute to this disease remain largely unknown.

Dysregulation of the hypothalamic pituitary adrenal (HPA) axis, a critical neuroendocrine system regulating responses to stressful stimuli, has been associated with mood disorders, including PPD^2^. HPA axis hyperactivity has been implicated in PPD as elevated levels of cortisol have been reported in patients^7,8^, although these observations are not always consistent^9^. Impairments in negative feedback suppression of the HPA axis have also been noted as women suffering from PPD exhibit decreased responsiveness to the dexamethasone suppression test^10^. The importance of maintaining an adequate level of peripartum HPA axis activity is further highlighted by the fact that under non-pathologic conditions the maternal HPA axis undergoes several adaptations. The normal diurnal rhythm of glucocorticoid secretion, cortisol in humans and corticosterone (CORT) in rodents, is altered so that levels remain flattened and more constant throughout the day^11^. In addition to this change in basal activity, the HPA axis response to a wide variety of stressors is attenuated^12–14^. As these maternal HPA axis adaptations are found in a variety of species, including rodents and humans, they are thought to be critical for preserving maternal mental health^15^.

Transcriptional regulation of corticotropin releasing hormone (CRH) serves as a focal point for modulating HPA axis activity^16^. Neurons in the paraventricular nucleus of the hypothalamus (PVN) release CRH, along with arginine vasopressin (AVP) and other neuropeptides with less robust adrenal regulatory activity, into the hypophyseal-portal circulation in response to stress or circadian stimuli. CRH and AVP stimulate downstream activation of the pituitary-adrenal axis, resulting in glucocorticoid secretion^17^. Of note, underlying the hyporeactive phenotype of the maternal HPA axis is proposed to be a decrease in transcription of CRH from the PVN^18^. Although rapid CRH transcription is essential for activation of the HPA axis, feedback modulation proves equally as important in order to prevent CRH overproduction and alteration of neuroendocrine and behavioral responses.

Transcriptional control of CRH by nuclear steroid hormone receptors has been described as a key mechanism for regulating CRH production and preventing excess HPA axis activity^19^. The corticosteroid receptors, glucocorticoid (GR) and mineralocorticoid receptors (MR), and the progesterone receptor (PR) have been localized to the PVN^20–22^. They have been found to modulate gene expression by binding to their respective ligand and triggering nuclear translocation to activate ligand-dependent transcription factors^23,24^. Nuclear receptor expression itself is regulated through diverse mechanisms, including autoregulation by their respective ligands and environmental insults such as stress^25^. Interestingly, glucocorticoids, estrogens, and progesterone are known to fluctuate dramatically during the peripartum period^26^. However, little is known about peripartum changes in the expression of steroid receptors at the level of the PVN and in response to gestational stress.

Rodent models have been devised to study the role stress plays in the development of PPD. Evidence gained through these models indicates peripartum stress results in the development of postpartum depressive-like behaviors^15,27–32^, often associated with anxiety^15,30^, and can lead to quantitative alterations in maternal care^15,27–33^. These changes are likely mediated by heightened CORT levels, as similar findings are reported after exogenous glucocorticoid administration during the peripartum period^34,35^. Abnormalities in maternal HPA axis circadian activity have also been noted following exposure to stress paradigms with psychosocial components^15,36^. Nevertheless, relevant brain molecular changes at different peripartum timepoints and in response to stressors more relevant to humans remain to be investigated.

Clinical studies have identified chronic exposure to mild to moderate psychosocial stress as a consistent predictor of PPD^4–6,37^. Here, we present a novel chronic psychosocial stress paradigm during pregnancy (CGS), which exposes pregnant mice to psychosocially challenging insults. Our CGS model relies on mild to moderate rodent psychosocial stressors which more closely capture the characteristics of stress experienced by human mothers and which are primarily processed through limbic regions, favoring activation of brain regions implicated in PPD^38^. Stressors are applied in an unpredictable fashion, hindering the development of adaptation mechanisms often associated with chronic stress paradigms^39^.

We find that exposure to CGS results in somatic changes in dams and offspring, postpartum maternal behavioral abnormalities, and dysregulation of the maternal HPA axis. To augment previous investigations on behavioral and endocrine effects of gestational stress we measured changes in CRH related signaling, as well as neuropeptides prominently expressed in stress responsive PVN neurons (AVP and oxytocin (OXT)), followed by changes in nuclear steroid hormone receptors known to regulate CRH. We report that altered PVN CRH signaling is associated with downregulation of GR, PR, and MR. These findings reaffirm the adverse effects of chronic psychosocial stress exposure during pregnancy on maternal behavior and neuroendocrine function, suggest that altered expression of steroid receptors is associated with HPA axis dysregulation and inappropriate maternal behavioral responses, and identify our novel CGS paradigm as a valuable model that will allow for further dissection of the molecular events that lead to PPD.

## Materials and methods

### Animals

Female C57BL6/J mice between 3 and 6 months of age were obtained from Jackson Laboratory and used for all experiments. Mice were initially housed in groups of four on a 14-h/10-h light–dark cycle (lights on 0600 h) with access to chow and water *ad libitum* and allowed to habituate for at least 14 days. Mice were divided by simple randomization into two experimental groups, control and CGS. Investigators were blinded to group allocations during neuroendocrine and behavioral tests. All animal procedures were approved by the Cincinnati Children’s Medical Center Animal Care and Use Committee and were in accordance with the National Institutes of Health guidelines.

### Chronic psychosocial stress paradigm during pregnancy (CGS)

**Fig. 1:**
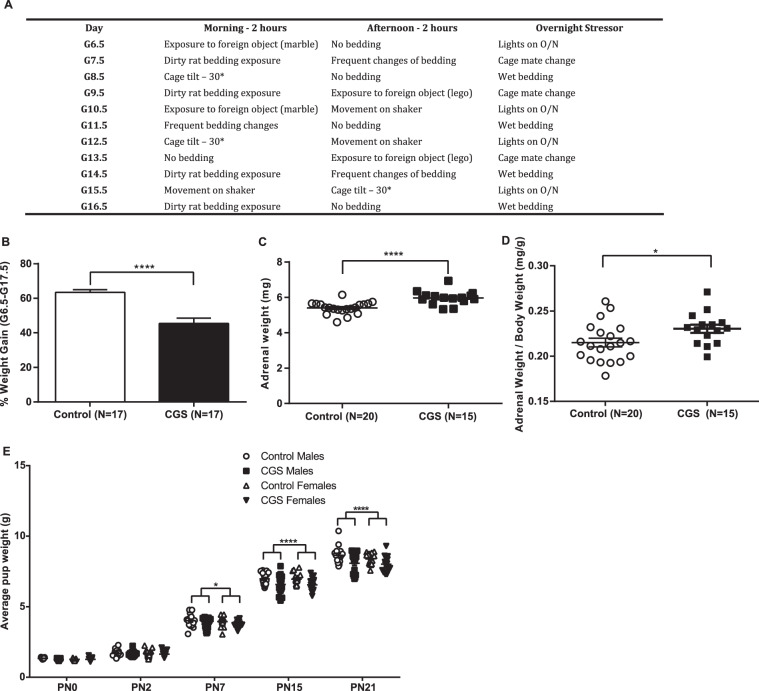
Chronic psychosocial stress during pregnancy leads to reductions in maternal and offspring body weight gain and increased maternal adrenal size. **a** Example of chronic psychosocial stress paradigm during pregnancy (CGS). **b** Maternal body weight change from G6.5 - G17.5, Control = 17, CGS = 17. **c** Raw maternal adrenal weights at PP2, Control = 20, CGS = 15. **d** Maternal adrenal weight/body weight ratio at PP2, Control = 20, CGS = 15. **e** Offspring body weight change from PN0 to PN21, Control = 17 litters, CGS = 17 litters. Data presented as mean + SEM. *p < .05, ****p < 0.0001, unpaired 2-tailed *t-*test for differences in maternal body weight and adrenal weight, mixed linear ANOVA for offspring body weight analysis with stress x sex x day model and litter as a randomized block factor. G, gestational day; PP, postpartum day; PN, postnatal day.

Females were set up for timed-matings at 1800 h, separated the following morning at 0800 h, and housed in groups of four. A copulatory plug marked 0.5 days post-coitum. From gestational day 6.5–16.5 (G6.5–G16.5), mice were exposed to variable psychosocial stressors (2 h each, 2 times per day, separated by at least a 2 h break, and overnight stressors). Day stressors included foreign object exposure, rat odor exposure, 30° cage tilt, bedding removal, and frequent bedding changes. Overnight stressors included cage mate change, wet bedding, and lights on (Fig. 1a). Control mice were left undisturbed. From G17.5, mice (CGS and control) were single-housed.

### Bodyweight

Body weight was measured on G6.5 and G17.5, and percentage body weight gain during gestation was calculated.

### Parturition, pregnancy outcomes, and litter characteristics

Pregnant female mice were monitored by observation from G17.5 until delivery. Cages were checked in the morning, afternoon, and night to calculate gestation length, determined by the timing of birth of the first pup. Number of pups in a litter, pup weights, and pup sex were recorded on the day of birth (postnatal day 0, PN0). Litters were culled to 3 male and 3 female pups per dam to ensure comparable conditions across all dams. Pups were weighed at different timepoints during the postnatal period (PN 2, 7, 15, 21).

### Serum corticosterone levels

Submandibular phlebotomy was performed at postpartum day 2 (PP2) in dams at nadir, peak, and 20-min after restraint stress. Serum corticosterone concentration was measured by ELISA per manufacturer’s protocols (BioVendor, Brno, Czech Republic; RTC002R).

### Adrenal gland measurements

Maternal adrenal glands were dissected at PP2, pruned of fat tissue, and weighed. Relative adrenal gland weights were analyzed.

### Behavioral tests

All behavioral tests were conducted during the early postpartum period, from postpartum day 2–8 (PP2–PP8), and testing proceeded from least to most stressful behavioral assessment. One cohort underwent testing for maternal behavior followed by forced swim test (FST) to measure depressive-like behavior. A separate cohort was used to assess locomotion in the open field test (OFT) and anxiety in the light/dark transition test (LD). A third cohort underwent sucrose preference test (SPT) followed by elevated zero maze (EZM) to evaluate anhedonia and confirm the presence of anxiety-like behaviors.

#### Maternal behavior assessment

Maternal behavior was evaluated following published protocols. From PP2–PP5, dams were habituated to the testing room for 5-min followed by a 30-min observation during the light cycle during which dams were assessed for the percentage of time spent licking/grooming pups, nursing, and off nest^40,41^. Fragmentation of maternal care was quantified by assessing the mean length of an individual licking/grooming bout and the total number of bouts^42^. Unpredictability in maternal behavior sequences and entropy rates were calculated as previously described^42–44^. Pup retrieval test was performed on PP6, as previously published^45^. Latencies to retrieve first pup and all pups were recorded in sec^45^. Nest quality was assessed on PP7, as previously described^46,47^.

#### Forced swim test (FST)

The FST was conducted, as previously described^48^, on PP8. Amount of time spent immobile, defined as lack of all movement except the minimal movement required to keep the mouse afloat, and frequency of immobility episodes were recorded.

#### Open field test (OFT)

The OFT was conducted on PP7, as previously described^49^. Motor activity was recorded for 1 h and data analyzed in 5-min intervals.

#### Light/dark transition test (LD)

The LD was conducted on PP8 as previously described^50^. The amount of time spent in each compartment was recorded over a 10-min period and started after being placed on the lighted side.

#### Sucrose preference test (SPT)

The SPT was conducted from PP0 to PP6, as previously described^51^. Preference was calculated using the averages from the last 4 days only as follows: preference % = [(sucrose consumption/sucrose + water consumption) × 100].

#### Elevated zero maze (EZM)

The EZM was conducted on PP8, as previously described^49^. Amount of time spent in the open arms was recorded over a 5-min period.

### RNA isolation and quantitative PCR

Brains were collected from pregnant females at G17.5, PP2, and PP7 under basal conditions, after CGS, and in virgin controls. Mice were sacrificed via cervical dislocations and brains were harvested, immediately frozen on dry ice, and stored at −80 °C. PVN micropunches were obtained from 60 µm thick frozen brain sections using a 1-mm puncher, as previously described^52^. Total RNA was extracted using the RNeasy Micro kit (Qiagen, Hilden, Germany; 74004) per manufacturer’s protocol. RNA was reverse transcribed using the Quantitect Reverse Transcriptase Kit (Qiagen, Hilden, Germany; 205310) according to the manufacturer’s protocols and cDNA was stored at −20 °C. qPCR was performed on each cDNA sample using Taqman system with Taqman Gene Expression Master Mix (ThermoFisher Scientific, Waltham, MA, USA). Gene expression data were calculated using the ΔΔCt method.

### Statistical analysis

Data were analyzed by Student’s *t*-test, one-way ANOVA followed by Tukey’s post hoc test, two-way ANOVA test followed by Sidak’s post hoc test (Prism 7.0c software; GraphPad Software, Inc., San Diego, CA, USA), or mixed linear factorial ANOVA with degrees of freedom calculated using the Kenward-Roger method (Proc Mixed, SAS version 9.4, SAS Institute, Cary, NC, USA) as indicated in figure legends. *P* < 0.05 was considered significant. The *n* represents either dams or litter numbers as indicated in figure legends. Results are reported as mean + standard error of the mean (s.e.m.).

## Results

### Effect of chronic psychosocial stress during pregnancy on maternal somatic parameters and litter characteristics

Relative to age-matched non-stressed controls, female mice exposed to CGS display a significant reduction in body weight gain during pregnancy (*t*-test, t_32_ = 5.256, *P* < 0.0001) (Fig. 1b). Similarly, we evaluated the effects of chronic stress on adrenal weights. Our data show CGS is associated with a significant increase in raw maternal adrenal weights (*t*-test, t_33_ = 4.582, *P* < 0.0001) (Fig. 1c) as well as adrenal weight/body weight ratio in the early postpartum period (*t*-test, t_33_ = 2.249, *P* = 0.031) (Fig. 1d). We further assessed the effects of CGS on litter characteristics. While no differences in offspring body weight were detected at birth, body weight was significantly reduced in male and female offspring from CGS dams compared with control offspring on PN 7 (post-hoc *P* < 0.05), PN15 (post-hoc *P* < 0.001), and PN21 (post-hoc *P* < 0.001) (F(4,212) = 5.480; *P* = 0.003 stress × day interaction) (Fig. 1e). There were no detectable differences in litter size (*t*-test, t_32_ = 1.437, *P* = 0.161) (Fig. S1a) or number of male and female pups per litter (F(1,64) = 0.179; P = 0.674 stress x sex interaction) (Fig. S1b). Likewise, there was no significant difference between dams exposed to CGS and age-matched controls for the length of gestation (*t*-test, t_32_ = 1.461, *P* = 0.154), total number of dead pups found (*t*-test, t_32_ = 0, *P* = 0.999), or number of dystocia events (*t*-test, t_32_ = 0.471, *P* = 0.641) (Table S1).

### Effect of chronic psychosocial stress during pregnancy on maternal behavior

**Fig. 2:**
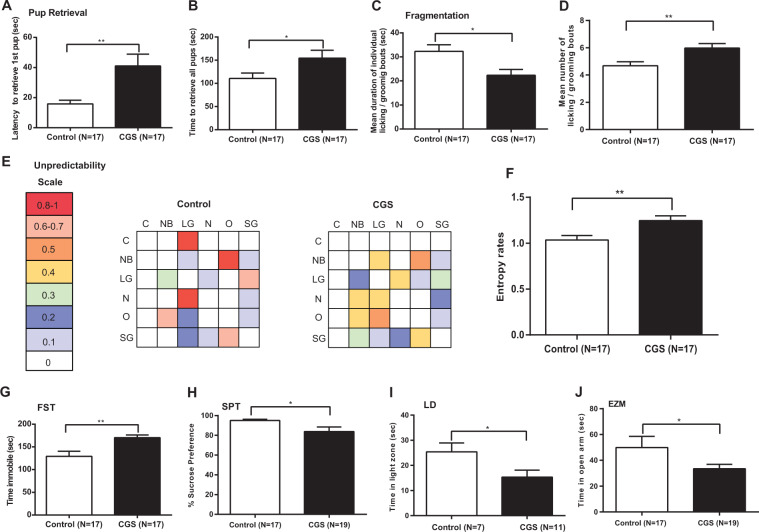
Chronic psychosocial stress during pregnancy leads to postpartum maternal behavioral deficits. Time to retrieve first pup (**a**) and all pups (**b**) into the nest in a pup retrieval task, Control = 17, CGS = 17. Mean duration (**c**) and number (**d**) of licking/grooming bouts recorded from postpartum day 2–5, and used to quantify fragmentation in maternal behavior, Control = 17, CGS = 17. **e** Matrix of conditional probabilities summarizing behavioral transitions for an individual dam where a color-coded scale is used to depict the likelihood that a particular form of maternal behavior is followed by another. **f** Entropy rates used to quantify degree of unpredictability in maternal behavior sequences, Control = 17, CGS = 17. **g** Time spent immobile in FST, Control = 17, CGS = 17. **h** Percent sucrose preference in SPT, Control = 17, CGS = 19. **i** Time spent in the light compartment in LD, Control = 7, CGS = 11. **j** Time spent in the open arm in EZM, Control = 17, CGS = 19. Data presented as mean + SEM *p < 0.05, **p < 0.01, unpaired 2-tailed *t*-test. C, carrying pups; NB, nest building; LG, licking/grooming pups; N, nursing; O, off pups; SG, self-grooming; FST, forced swim test; SPT, sucrose preference test; LD, light/dark transition test; EZM, elevated zero maze.

To assess whether exposure to CGS is associated with impairments in parenting behavior, we examined maternal care in the early postpartum period in CGS dams and age-matched non-stressed controls. Analysis of maternal care behavior in the pup retrieval task revealed CGS dams displayed a significant increase in latency to retrieve the first pup from the opposite corner of the home cage into the nest (*t*-test, t_32_ = 3.071, *P* = 0.004) (Fig. 2a), as well as an increased time to retrieve all pups to the nest (*t*-test, t_32_ = 2.097, *P* = 0.044) (Fig. 2b) suggesting deficits in maternal care. We further examined the quantity and quality of maternal care delivered by CGS and control dams. No differences in percent of time spent grooming pups (*t*-test, t_32_ = 0.908, *P* = 0.371) (Fig. S2a), nursing pups (*t*-test, t_32_ = 1.534, *P* = 0.135) (Fig. S2b), or time off nest (*t*-test, t_32_ = 0.629, *P* = 0.533) (Fig. S2c) were observed between the two conditions, suggesting overall maternal care duration to be consistent. Likewise, there were no detectable differences when assessing nest quality between the two groups (*t*-test, t_32_ = 0.899, *P* = 0.375) (Fig. S2d). In addition to these typical measures of maternal care, we analyzed more qualitative aspects of maternal behavior after CGS exposure, including the degree of fragmentation and unpredictability of maternal signals. Fragmented and unpredictable care patterns seem to reflect poorer maternal care quality and have been associated with maternal pathology^42–44,53–55^. For CGS dams, the mean duration of licking/grooming bouts was significantly shorter than control dams (*t*-test, t_32_ = 2.709, *P* = 0.011) (Fig. 2c) and was accompanied with a significant increase in the average number of bouts (*t*-test, t_32_ = 2.904, *P* = 0.007) (Fig. 2d), suggesting CGS exposure increases fragmentation of care, resulting in numerous short episodes of nurturing behavior. To measure the degree of unpredictability in maternal behavioral sequences, we generated a matrix of conditional probabilities depicting the proportion of time one aspect of maternal behavior is followed by another^42,43^. Interestingly, CGS dams displayed mid-range (green–orange) probabilities of following a given sequence of maternal behaviors as opposed to control dams which tended to have high (pink-red) or low (blue) probabilities (Fig. 2e), suggesting CGS exposure results in a more inconsistent, unpredictable pattern of care. Entropy rates, used to quantify this degree of randomness, were significantly higher in CGS dams than controls (*t*-test, t_32_ = 2.926, *P* = 0.006) (Fig. 2f). Following assessments for maternal behavior, mice in the study were exposed to FST as a way to evaluate despair-related behavior. CGS dams displayed a significant increase in the amount of time spent immobile (*t*-test, t_32_ = 3.297, *P* = 0.002), suggesting a depressive-like phenotype (Fig. 2c). In a separate experiment, we evaluated the presence of anhedonia, a core symptom of depressive behavior often seen in PPD^37^, by allowing dams to freely choose between 4% sucrose solution or water. CGS dams had a significantly reduced preference for 4% sucrose solution than control dams (*t*-test, t_34_ = 2.200, *P* = 0.035) (Fig. 2h), suggesting anhedonic behavior. We also evaluated the effects of CGS on anxiety-related behavior via LD and EZM. CGS dams spent significantly less time in the light area of the LD apparatus (F(1,28.4) = 5.09, *P* = 0.032) (Fig. 2i) and less time in the open arms of EZM (*t*-test, t_34_ = 1.828, *P* = 0.038) (Fig. 2j) compared with controls, indicating increased anxiety in CGS dams in the early postpartum period (Fig. 2d). No differences were observed between dams exposed to CGS and controls in the number of immobility episodes in the FST (*t*-test, t_32_ = 0.496, P = 0.917) (Fig. S3a), total locomotor activity in OFT (F(11, 283) = 0.85; *P* = 0.591 stress × interval interaction) (Fig. S3b), or number of entries into open arms of EZM (*t*-test, t_34_ = 1.001, *P* = 0.983) (Fig. S3c), suggesting no differences in locomotor parameters.

### Effect of chronic psychosocial stress during pregnancy on maternal neuroendocrine function

**Fig. 3:**
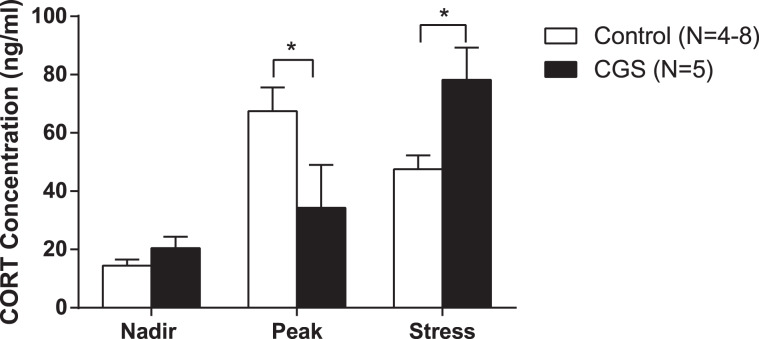
Chronic psychosocial stress during pregnancy leads to postpartum maternal HPA axis dysregulation. Serum CORT levels measured at nadir, peak, and after 20 min of restraint stress on PP2, Control = 4–8 per time point, CGS = 5 per time point. Data presented as mean + SEM. *p < 0.05, two-way ANOVA followed by Sidak’s post hoc test. CORT, corticosterone.

To evaluate effects of CGS on maternal neuroendocrine function, we measured circadian and acute stress-induced secretion of serum CORT levels at PP2. We found a significant reduction in serum CORT levels during the circadian peak timepoint in dams exposed to CGS when compared with age-matched controls (F(2,29) = 7.585; *P* = 0.002 time × stress interaction; post-hoc *P* < 0.05), although no changes were detected at nadir (Fig. 3). Following 20-min of restraint stress, serum CORT levels were significantly elevated in dams exposed to CGS as compared to control dams (post-hoc *P* < 0.05) (Fig. 3).

### Effect of chronic psychosocial stress during pregnancy on hypothalamic molecular regulators of the HPA axis

**Fig. 4:**
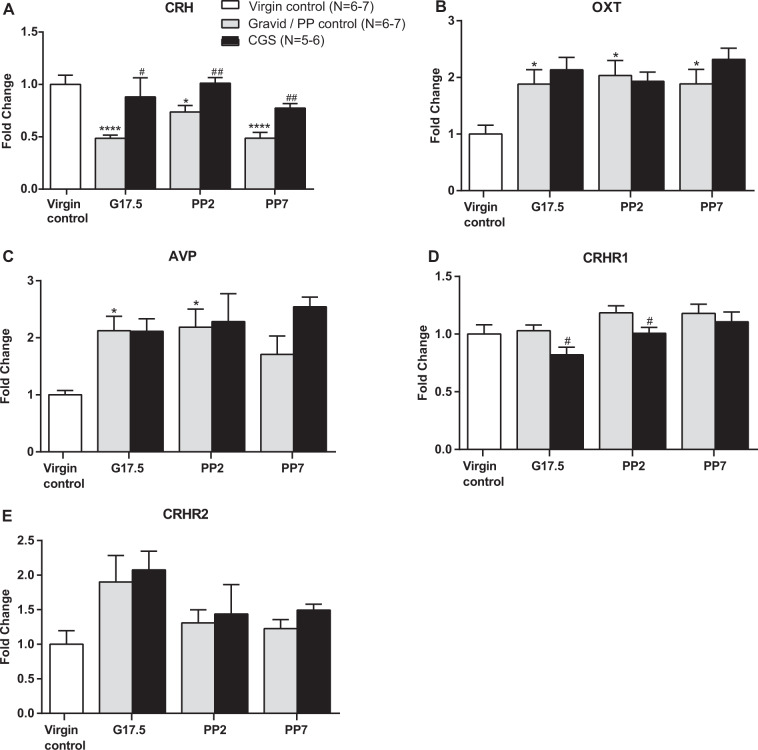
Chronic psychosocial stress during pregnancy is associated with dysregulation in CRH signaling pathway. **a** In the PVN of gravid/postpartum control mice, there is a downregulation of CRH expression from G17.5 to PP7 which is prevented by CGS. **b** AVP and **c** OXT are increased in with gravid/postpartum control mice on G17.5 to PP7 and G17.5 to PP2, respectively, and no changes in expression are seen after CGS. **d** A downregulation of CRHR1 expression is observed on G17.5 and PP2 after CGS. **e** No differences in CRHR2 expression were observed, Virgin control = 6–7, gravid / PP control = 6–7 per time point, CGS = 5–6 per time point. Data presented as mean + SEM. *p < 0.05, ****p < 0.0001, one-way ANOVA followed by Tukey’s post hoc test for differences from virgin control. ^#^*p* < 0.05, ^##^*p* < 0.01, unpaired 2-tailed *t*-test for differences within a time point be*t*ween CGS and gravid/ PP control. OXT, oxytocin; CRHR1, CRH receptor 1; CRHR2, CRH receptor 2.

To determine whether the observed changes in maternal neuroendocrine function in response to CGS may be mediated by alterations in key hypothalamic molecular regulators of the HPA axis, we measured changes in PVN CRH, OXT, and AVP via qPCR. In an initial analysis, gravid/postpartum control mice were compared with virgin controls with a one-way ANOVA. We observed a significant downregulation in CRH mRNA expression in female mice on G17.5 (post-hoc *P* < 0.0001), PP2 (post-hoc *P* < 0.05), and PP7 (post-hoc *P* < 0.0001) when compared with virgin controls (one-way ANOVA F(3,23) = 15.16; *P* < 0.0001) (Fig. 4a). In contrast, OXT is significantly increased in gravid/postpartum control mice from G17.5 to PP7 (post-hoc *P* < 0.05) relative to virgin controls (one-way ANOVA F(3,22) = 4.899; *P* = 0.009) (Fig. 4b). Our data also show a significant upregulation of AVP in female mice during the peripartum period, from G17.5 to PP2 (post-hoc *P* < 0.05), when compared with virgin controls (one-way ANOVA F(3,22) = 3.9021; *P* = 0.022) (Fig. 4c).

Comparison of the CGS mice with gravid/postpartum control mice was then analyzed. Interestingly, we found CGS had a significant effect on CRH mRNA expression in the PVN where it was associated with a significant increase in CRH on G17.5 (*t*-test, t_11_ = 2.301, *P* = 0.042), PP2 (*t*-test, t_11_ = 3.281, *P* = 0.007), and PP7 (*t*-test, t_9_ = 4.032, *P* = 0.003) (Fig. 4a). However, no detectable changes where observed in OXT mRNA (Fig. 4b) or AVP mRNA levels (Fig. 4c) in the PVN after CGS exposure compared with gravid/postpartum controls.

To further evaluate the effects of CGS on the CRH signaling pathway, we measured changes in expression levels of CRH receptors type 1 (CRHR1) and 2 (CRHR2). No differences were observed in CRHR1 mRNA (Fig. 4d) and CRHR2 mRNA (Fig. 4e) between control pregnant females at G17.5 or different timepoints in the postpartum period when compared with virgin controls. However, exposure to CGS resulted in downregulation of CRHR1 at G17.5 (*t*-test, t_10_ = 2.589, *P* = 0.027) and PP2 (*t*-test, t_12_ = 2.204, *P* = 0.048) (Fig. 4d). By contrast, there was no effect of CGS on CRHR2 expression (Fig. 4e).

### Effects of chronic psychosocial stress during pregnancy on nuclear steroid hormone receptors associated with modulation of CRH signaling and HPA axis function

**Fig. 5:**
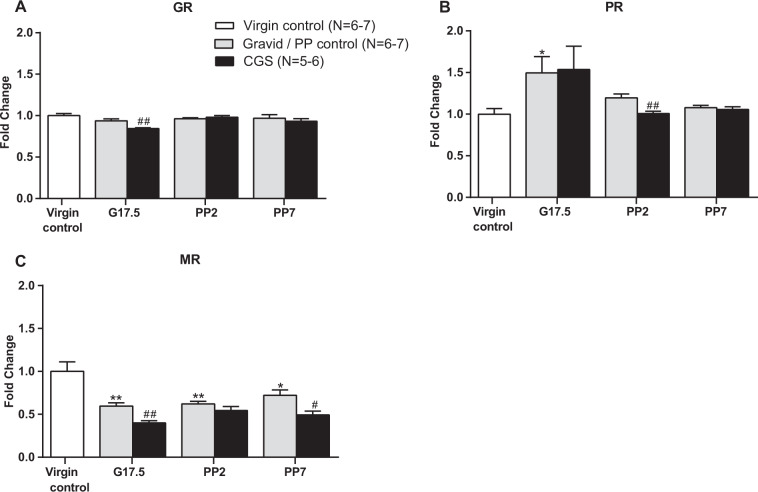
Chronic psychosocial stress during pregnancy is associated with downregulation of nuclear steroid hormone receptors linked to CRH regulation. **a** In the PVN, there is a downregulation of GR expression in pregnant females at G17.5 after CGS. **b** PR is increased at G17.5 in gravid control mice and downregulated at PP2 in CGS animals. **c** MR is decreased in gravid/postpartum control mice from G17.5 to PP7. CGS is associated with a further downregulation in expression of MR on G17.5 and PP7, Virgin control = 6–7, gravid/PP control = 6–7 per time point, CGS = 5–6 per time point. Data presented as mean + SEM. *p < 0.05, **p < 0.01, one-way ANOVA followed by Tukey’s post hoc test for differences from virgin control. ^#^*p* < 0.05, ^##^*p* < 0.01, unpaired 2-tailed *t*-test for differences within a time point be*t*ween CGS and gravid / PP control. GR, glucocorticoid receptor; PR, progesterone receptor; MR, mineralocorticoid receptor.

To determine the mechanism by which CGS leads to increased CRH in the PVN, we measured changes in expression of GR, PR, and MR, nuclear steroid hormone receptors responsible for CRH regulation. Our data show that although GR mRNA expression was not altered by pregnancy, CGS had a significant effect on GR where it was associated with a decrease in expression on G17.5 (*t*-test, t_11_ = 3.566, *P* = 0.004) (Fig. 5a). We observed an increase in PR mRNA expression in control gravid mice on G17.5 (post-hoc *P* < 0.05) when compared with virgin controls (one-way ANOVA F(3,22) = 3.703; *P* = 0.0269) (Fig. 5b). Exposure to CGS did not affect this pregnancy related increase in PR but was found to downregulate PR mRNA levels on PP2 (*t*-test, t_12_ = 3.433, *P* = 0.005) (Fig. 5b). In contrast, MR is significantly downregulated from G17.5 to PP7 in control gravid mice (post-hoc *P* < 0.01) relative to virgin controls (one-way ANOVA F(3,22) = 8.145; *P* = 0.008) (Fig. 5c). CGS was associated with a further reduction in MR mRNA levels on G17.5 (*t*-test, t_11_ = 4.150, *P* = 0.002) and PP7 (*t*-test, t_9_ = 2.793, *P* = 0.021) (Fig. 5c).

## Discussion

In the current study, we find that our novel CGS paradigm results in physiological changes in dams and offspring, as well as abnormal behaviors in the early postpartum period, including fragmented and unpredictable maternal care patterns, and the development of depression and anxiety-like phenotypes. Functionally, we find these changes are correlated with disrupted neuroendocrine activity, including alterations in the circadian rhythm of glucocorticoid secretion and HPA axis hyperreactivity in response to a novel stressor. We further observe selective changes in the CRH signaling pathway after maternal stress, including increased PVN CRH mRNA and downregulation of CRHR1 mRNA. We find these changes are associated with decreased mRNAs of GR, PR, and MR, suggesting nuclear steroid hormone receptors may underly changes in HPA axis and behavior.

Our data indicate CGS leads to maternal and offspring somatic changes. Exposure to CGS reduced maternal body weight gain during pregnancy and decreased offspring weight gain during postnatal development. Interestingly, offspring weight differences were not detected at birth, suggesting CGS did not disrupt placental nutrient transfer capacity essential for fetal growth^56^. Despite decreased postnatal weight in prenatally stressed offspring, we did not detect differences in amount of time spent nursing in CGS and control dams, suggesting weight reduction might reflect alterations in offspring metabolic systems. Altered maternal glucocorticoid levels might play a role in mediating these effects as other studies have shown offspring weight gain is attenuated after in-utero exposure to exogeneous CORT^57^. Mechanisms that help protect against elevated maternal glucocorticoids include dampening of the maternal HPA axis and placental expression of 11β-hydroxysteroid dehydrogenase 2 (11βHSD2), an enzyme that converts glucocorticoids into inactive metabolites^58^. CGS exposure seems to prevent maternal HPA axis attenuation and could have modified placental 11βHSD2 function, although this remains to be tested. Importantly, no differences were observed in gestational length between CGS and control dams, suggesting our maternal stress model did not interfere with mechanisms important for parturition^59^. Furthermore, no differences were seen in litter size or offspring sex per litter, indicating CGS exposure did not result in implantation failure or fetal resorptions.

In this study, we also find that CGS leads to abnormalities in maternal behavior during the early postpartum period. Fragmented and unpredictable maternal care patterns emerged following CGS, although the total duration of maternal behaviors were not affected. Exposure to CGS also resulted in the development of anxiety, anhedonia, and depressive-related behavior. These results are consistent with human data where some of the core features of women suffering from PPD include interrupted patterns of maternal care^60^, lack of child attachment, and depressive mood^61^. Inconsistent maternal care patterns have been hypothesized to result from conflict that arises at the inability of dams to respond to pups’ specific needs during active maternal interactions^62^, a process which is thought to depend on the mesolimbic dopaminergic system^63^. CGS dams also exhibit deficits in pup retrieval, a unique element of maternal behavior that relies on goal-directed maternal responses thought to be controlled by neural connections between the medial preoptic area and the mesolimbic dopaminergic system^64^. It is possible our paradigm could be causing disruptions in limbic stress regulatory pathways^65^ that overlap with regions critical for processing pup-related inputs and modulating goal-directed maternal responsiveness, leaving more reflexive brainstem dependent responses, such as nursing^66^, unaffected. Importantly, as no differences were observed in indices of locomotion following CGS, behavioral abnormalities measured in our study were not confounded by overall changes in ambulation.

Our findings further demonstrate that CGS disrupts adaptive changes in maternal neuroendocrine systems by altering the activity of the maternal HPA axis. We show that CGS dams hypersecrete CORT in response to a novel stressor in the early postpartum period relative to controls. They also exhibit increased adrenal gland weights, consistent with increased production of adrenal glucocorticoids following chronic drive of the HPA axis. The mechanisms of this higher stress responsivity may be related to the loss of glucocorticoid feedback control of the HPA axis, which could be associated with the decrease in GR observed in the PVN and potentially in other areas, such as the hippocampus^48^. In addition, stress exposure has been shown to affect levels of reproductive hormones, and their neurosteroid metabolites^67–69^. As these are known to modulate HPA axis function, decreased levels of these hormones in our model may also underlie the observed reduced negative feedback on the HPA axis. Importantly, we hypothesize brief periods of pup separation during neuroendocrine and behavioral assays in our study did not significantly contribute to our stress paradigm. Previous studies have shown brief pup separation is not a sufficient stressor to increase maternal HPA axis activity or result in depressive-like behaviors during mid-to-late lactation^27,70–73^. Nevertheless, future studies are needed to determine the precise effect of pup removal on early postpartum neuroendocrine activity and behavior.

Interestingly, despite stress-induced hyper-reactivity of the HPA axis, basal CORT production was attenuated as seen by decreased levels during the peak phase of the diurnal rhythm of CORT secretion. These results could be explained by decreased PVN MR levels observed, which have been postulated to regulate basal CORT production by virtue of its high affinity – low occupancy pattern of expression^74^.

Our data further indicate that during the peripartum period, PVN OXT and AVP mRNA levels are upregulated, while PVN CRH mRNA levels are decreased. These results are consistent with previous studies^14,15^ and thought to be necessary for the onset and maintenance of maternal behavior. In the current study, we find that CGS increased peripartum PVN CRH mRNA expression and downregulated CRHR1 mRNA, suggesting elevated peripartum CRH signaling underlie dysregulations in postpartum maternal HPA axis and behavior. These changes were selective to the CRH signaling pathway, as OXT and AVP levels were undisturbed after CGS exposure. A number of extrahypothalamic sites express CRH, including the extended amygdala, hippocampus, and brainstem^75^. Of particular interest is CRH production in the central nucleus of the amygdala (CeA), where it has been implicated in regulating behavioral responses to anxiety and depression^76–78^. Synthesis and secretion of CRH in the CeA are key steps in the stress response, as CeA CRH expression increases after chronic stress and exerts a stimulatory influence on the HPA axis^79^. It remains to be assessed if in our model CeA CRH expression may be modulating behavioral changes, via its effect on HPA axis function or its neuromodulatory capacity on target neurons^80^. Our findings of decreased CRHR1 could be interpreted as being adaptive to protect from neurotoxic or metabolic challenges related to excessive CRH signaling^81^. In humans, increased levels of CRH have been reported in PPD^76^ and been suggested as a possible diagnostic criterion for the disease^82^.

Interestingly, increased peripartum PVN CRH signaling after CGS exposure was associated with a downregulation in nuclear steroid hormone receptors GR, PR, and MR. These receptors function as transcription factors, regulating the expression of a diverse set of genes, including CRH, upon ligand-dependent activation and homo or heterodimerization^23,83^. Although, a classic steroid hormone response element has not been identified in the CRH gene promoter, there have been several regions within the promoter described as binding sites for these nuclear steroid receptors. These receptors can also regulate CRH transcription by binding other transcription factors via protein-protein interactions and modulating additional signaling pathways^84–87^. Thus, it is possible that PVN changes in GR, PR, and MR could be underlying the upregulation in CRH mRNA and enhanced capacity for stress excitation observed in our model after CGS exposure. Alternatively, our CGS paradigm could be altering brainstem noradrenergic neurons known to provide direct excitatory input to neurons in the PVN inducing CRH gene transcription through α1 adrenergic receptor activation^88^. Future studies employing viral-based delivery of cre-recombinase for selective spatiotemporal modulation of nuclear steroid hormone receptors are required to dissociate the mechanistic role of GR, PR, and MR in mediating abnormalities in HPA axis function and behavior during the postpartum period.

Our results are consistent with previously published findings and further demonstrate that chronic psychosocial stress during pregnancy can affect qualitative aspects of maternal care, resulting in fragmented and unpredictable care patterns. Erratic maternal care patterns have been previously reported in dams subjected to impoverished/limited bedding environment^42–44^ and have been linked to emotional and cognitive disturbances in rodent and human offspring^42,43,53–55^. We also find psychosocial stressors contribute to the emergence of PPD by interfering with basal and stress-induced peripartum HPA axis adaptations. A recent study found chemogenetic stimulation of PVN CRH neurons to be sufficient to generate deficits in maternal care as well as depressive-like behaviors^89^. Consistent with these findings, our results implicate increased PVN CRH signaling with the emergence of abnormal postpartum behaviors and neuroendocrine function, and further suggest downregulation in PVN nuclear steroid receptors could in part be mediating these effects.

In conclusion, we present a novel animal model based on chronic psychosocial stress exposure during pregnancy that exhibits abnormal postpartum behaviors, HPA axis hyperresponsiveness, and enhanced CRH signaling, similar to what has been reported in a subset of women with PPD. To our knowledge, this is the first study implicating peripartum alterations in nuclear steroid hormone receptors known to regulate CRH transcription in the pathogenesis of PPD. Our rodent model could provide a unique tool for testing the effects of pharmacological manipulation of these receptors on HPA axis function and mood regulation, thus allowing for the development of novel therapeutic approaches. Additionally, examining detailed mechanisms linking altered glucocorticoid levels to behavioral dysregulation in the postpartum period, such as effects on the structural plasticity of stress neural circuits, warrants further investigation. Finally, performing RNA sequencing in this model to generate transcriptional profiles in brain regions known to play essential roles in the regulation of emotional liability and neuroendocrine function could provide opportunities for understanding neurobiological pathways that become dysregulated following psychosocial stress, thus potentially informing strategies to diagnose and treat PPD.

## Supplementary information

Supplementary Information

Supplementary Figure Table S1

Supplementary Figure 1

Supplementary Figure 2

Supplementary Figure 3
