# Supplementary material for: Chronic psychosocial stress during pregnancy affects maternal behavior and neuroendocrine function and modulates hypothalamic CRH and nuclear steroid receptor expression
Source: Transl Psychiatry. 2020 Jan 16;10:6. doi: 10.1038/s41398-020-0704-2 (PMC7026416; doi:10.1038/s41398-020-0704-2)
Supplement: Supplementary file 1 — Supplementary Information [file 41398_2020_704_MOESM1_ESM.docx]

**Supplementary Information:**

**Supplementary Material and methods:** Complete description

**Supplementary figure legends:**

Supplementary Table 1: Pregnancy and litter characteristics.

Supplementary Figure 1: Chronic psychosocial stress during pregnancy has no effect on litter size or offspring sex per litter.

Supplementary Figure 2: Undisturbed measures of maternal care in the early postpartum period after chronic psychosocial stress during pregnancy.

Supplementary Figure 3: Chronic psychosocial stress has no effect on indices of movement.

**Supplementary Material and methods**

**Animals** Female C57BL6/J mice between 3 and 6 months of age were obtained from Jackson Laboratory and used for all experiments. Mice were initially housed in groups of four on a 14-hour/10-hr light-dark cycle (lights on 0600 h) with access to chow and water *ad* *libitum* and allowed to habituate for at least 14 days. Mice were divided by simple randomization into two experimental groups, control and CGS. Investigators were blinded to group allocations during neuroendocrine and behavioral tests. Five different cohorts were used for this study. The first cohort was used to record pregnancy related parameters and maternal and offspring characteristics followed by maternal care assessments and forced swim test (FST) (control n=17, CGS n=17). The second cohort was used for open field test (OFT) and a subset of these mice were tested on the light/dark transition test (LD) (control n=14, CGS n=19). The third cohort was used for CORT measurements and adrenal gland weights (control n=20, CGS n=15). The fourth cohort was used to measure gene expression changes by qPCR (control n=20, CGS n=17). The fifth cohort was used for sucrose preference test (SPT) followed by elevated zero maze (EZM) (control n=17, CGS n=19). All animal procedures were approved by the Cincinnati Children’s Medical Center Animal Care and Use Committee and were in accordance with the National Institutes of Health guidelines.

**Chronic psychosocial stress paradigm during pregnancy (CGS)** Females were set up for timed-matings at 1800h, separated the following morning at 0800h, and housed in groups of four. A copulatory plug marked 0.5 days post-coitum. From gestational day 6.5 to 16.5 (G6.5-G16.5), mice were exposed to variable psychosocial stressors (2h each, 2 times per day, separated by at least a 2h break, and overnight stressors). Day stressors included foreign object exposure, rat odor exposure, 30⁰ cage tilt, bedding removal, and frequent bedding changes. Overnight stressors included cage mate change, wet bedding, and lights on (Fig. 1a). Control mice were left undisturbed. From G17.5, mice (CGS and control) were single-housed.

**Bodyweight** Body weight was measured on G6.5 and G17.5, and percentage body weight gain during gestation was calculated. The weight on the first day of CGS paradigm (G6.5) was set as 100% and the percentage of body weight gained on G17.5 was analyzed.

**Parturition, pregnancy outcomes, and litter characteristics** Pregnant female mice were monitored by observation from G17.5 until delivery. Cages were checked in the morning, afternoon, and night to calculate gestation length, determined by the timing of birth of the first pup. Number of pups in a litter, pup weights, and pup sex were recorded on the day of birth (postnatal day 0, PN0). Litters were culled to 3 male and 3 female pups per dam to ensure comparable conditions across all dams. Pups were weighed at different timepoints during the postnatal period (PN 2, 7, 15, 21). Adult females were excluded from the study if the female was in labor for longer than 12 hours or presented with dystocia.

**Serum corticosterone levels** Submandibular phlebotomy was performed at postpartum day 2 (PP2) in dams at nadir, peak, and 20-min after restraint stress. Maternal blood was collected in serum separator tubes, centrifuged at 21,130 x g for 6-min, and the serum was removed and stored at -20⁰C. Serum corticosterone concentration was measured by ELISA per manufacturer’s protocols (BioVendor, Brno, Czech Republic; RTC002R).

**Adrenal gland measurements** Maternal adrenal glands were dissected at PP2, pruned of fat tissue, and weighed. Relative adrenal gland weights (total weight of right plus left adrenals/body weight in milligrams per gram) were analyzed.

**Behavioral tests** All behavioral tests were conducted during the early postpartum period, from postpartum day 2 to 8 (PP2-PP8), and testing proceeded from least to most stressful behavioral assessment. Three separate cohorts were used. One cohort underwent testing for maternal behavior followed by forced swim test (FST) to measure depressive-like behavior. A separate cohort was used to assess locomotion in the open field test (OFT) and anxiety changes in the light/dark transition test (LD) at the Cincinnati Children’s Research Foundation’s Animal Behavior Core. A third cohort underwent sucrose preference test (SPT) followed by elevated zero maze (EZM) to evaluate anhedonia and confirm the presence of anxiety-like behaviors.

*Maternal behavior assessment* Maternal behavior was evaluated following published protocols. Briefly, from PP2-PP5, dams were habituated to the testing room for 5-min followed by a 30-min observation during the light cycle during which dams were assessed for the percentage of time spent licking/grooming pups, nursing, and off nest^1,2^. Fragmentation of maternal care was quantified by assessing the mean length of an individual licking/grooming bout and the total number of bouts^3^. Unpredictability in maternal behavior sequences and entropy rates were calculated as previously described^3-5^. Dams were identified as performing one of six different behaviors: carrying pups (C), nest building (NB), licking/grooming pups (LG), nursing (N), off-pups (O), or self-grooming (SG). Only one behavior at a given time was possible. The conditional probabilities of each dam displaying a sequence of behaviors were calculated using an empirical transition matrix <p_ij_> _i,j=1...M_ where p_ij_ measures the proportion of times the dam was recorded as transitioning from behavior *i* to behavior *j* and M=6 is the total possible number of behaviors^3,4^. The transition matrix was used to visualize transition probabilities where each row represents a starting behavior and each column the behavior being transitioned into. Behavioral sequences were then modeled by a first-order Markov chain and the entropy rate of the process was calculated^3,4^. Entropy was first calculated for each row of the transition matrix and row entropy values were combined into a single entropy rate measure using $E=-\sum_{i=1}^{M} \pi_{i}\sum_{j=1}^{M} p_{ij}log\left( p_{ij} \right)$, where the stationary distribution $\pi_{i}$ of the Markov chain was defined as the proportion of time a dam was exhibiting each behavior^3,4^. As such, the entropy rate becomes a weighted average of row entropy values and represents the degree of randomness in maternal behavior sequences^3,4^. Pup retrieval test was performed on PP6, as previously published^6^. Dams were removed from the home cage and the pups were scattered randomly on the opposite side of the cage. The dam was returned to the original nest position and given 10-min to retrieve pups to the nest. Latencies to retrieve first pup and all pups were recorded in sec^6^. Nest quality was assessed on PP7, as previously described^7,8^.

*Forced swim test (FST)* The FST was conducted, as previously described^9^, on PP8. Briefly, mice were placed in a 2 L beaker with 1.4 L of water at room temperature. Mice were continuously monitored for immobility from 2 to 6-min in 6-min trial. Amount of time spent immobile, defined as lack of all movement except the minimal movement required to keep the mouse afloat, and frequency of immobility episodes were recorded.

*Open field test (OFT)* The OFT was conducted on PP7, as previously described^10^. Briefly, mice were placed in an automated locomotor activity chamber (41 cm x 41 cm x 38 cm) (San Diego Instruments, San Diego) with 16 photobeams spaced 2.5 cm apart in the x and y planes. Motor activity was recorded for 1h and data analyzed in 5-min intervals.

*Light/dark transition test (LD)* The LD was conducted on PP8 as previously described^11^. Briefly, mice were placed inside the activity chamber used for OFT which included a black acrylic insert that divided the chamber in half so that one side was light (20.5 x 41 cm) and the other dark (20.5 x 41 cm) with a 7 x 7 cm opening between them. Amount of time spent in each compartment was recorded over a 10-min period and started after being placed on the lighted side.

*Sucrose preference test (SPT)* The SPT was conducted from PP0 to PP6, as previously described^12^. Briefly, dams were housed with ad libitum food and given free access to one 100 ml bottle of 4% sucrose solution and another 100 ml bottle of tap water. Sucrose and water consumption (ml) were measured and the placement of the bottles was interchanged daily to reduce any confound introduced by side-bias. Preference was calculated using the averages from the last 4 days only as follows: preference % = [(sucrose consumption/sucrose + water consumption) × 100].

*Elevated Zero Maze (EZM****)*** The EZM was conducted on PP8, as previously described^10^. Amount of time spent in the open arms was recorded over a 5-min period.

**RNA isolation and quantitative PCR** Brains were collected from pregnant females at G17.5, PP2, and PP7 under basal conditions, after CGS, and in virgin controls. Mice were sacrificed via cervical dislocations and brains were harvested, immediately frozen on dry ice, and stored at -80⁰C. PVN micropunches were obtained from 60 µm thick frozen brain sections using a 1-mm puncher, as previously described^13^. Total RNA was extracted using the RNeasy Micro kit (Qiagen, Hilden, Germany; 74004) per manufacturer’s protocol. RNA was reverse transcribed using the Quantitect Reverse Transcriptase Kit (Qiagen, Hilden, Germany; 205310) according to manufacturer’s protocols and cDNA was stored at -20⁰C. qPCR was performed on each cDNA sample using Taqman system with Taqman Gene Expression Master Mix (ThermoFisher Scientific, Waltham, MA, USA). Specific probes were used to quantify murine CRH (Mm04206019_m1, ThermoFisher Scientific), CRHR1 ([Mm00432670_m1, ThermoFisher Scientific),](https://www.thermofisher.com/taqman-gene-expression/product/Mm00432670_m1?CID=&ICID=&subtype=ge_all)CRHR2 (Mm00438308_m1, ThermoFisher Scientific), AVP ([Mm00437761_g1](https://www.thermofisher.com/taqman-gene-expression/product/Mm00437761_g1?CID=&ICID=&subtype=), ThermoFisher Scientific), OXT (Mm01329577_g1, ThermoFisher Scientific), GR (Mm00433832_m1, ThermoFisher Scientific), PR (Mm00435628_m1, ThermoFisher Scientific), MR (Mm01241596_m1, ThermoFisher Scientific), and GAPDH (Mm99999915_g1, ThermoFisher Scientific) as endogenous control. 6 ng of cDNA template was used per well, and samples were run in duplicates. qPCR reactions were run on an Applied Biosystems StepOnePlus Real-Time PCR instrument (Applied Biosystems, Foster City, CA, USA). Gene expression data were calculated using the ΔΔCt method.

**Statistical analysis** The number of animals per experiment was based on a power analysis, calculated using standard algorithms^14^ with reference to our own pilot data and previously published data. Data were analyzed by Student’s *t* test, one-way ANOVA followed by Tukey’s post hoc test, two-way ANOVA test followed by Sidak’s post hoc test (Prism 7.0c software; GraphPad Software, Inc., San Diego, CA, USA), or mixed linear factorial ANOVA with degrees of freedom calculated using the Kenward-Roger method (Proc Mixed, SAS version 9.4, SAS Institute, Cary, NC, USA) as indicated in figure legends. P<0.05 was considered significant. The n represents either dams or litter numbers as indicated in figure legends. Results are reported as mean + standard error of the mean (s.e.m.).

**Supplementary figure legends**

**Supplementary Table 1. Pregnancy and litter characteristics.** No differences were observed between control and CGS groups for length of pregnancy, number of dystocia events, and number of dead pups per litter, Control = 17, CGS = 17, unpaired 2-tailed *t* test.

**Supplementary Figure 1. Chronic psychosocial stress during pregnancy has no effect on litter size or offspring sex per litter.** Litter size (**a**) and number of male and female pups per litter (**b**) were recorded at birth, Control = 17 litters, CGS = 17 litters. Data presented as mean + SEM. Unpaired 2-tailed *t* test for litter size or two-way ANOVA for offspring sex per litter.

**Supplementary Figure 2. Undisturbed quantitative measures of maternal care in the early postpartum period after chronic psychosocial stress during pregnancy.** Percent time spent grooming pups (**a**) nursing pups (**b**) and off nest (**c**) were measured during the early postpartum period, from postpartum day 2 to 5, Control = 17, CGS = 17. (**d**) Assessment of quality of the nest built, Control = 17, CGS = 17. Data presented as mean + SEM. Unpaired 2-tailed *t* test.

**Supplementary Figure 3. Chronic psychosocial stress has no effect on indices of movement.** (**a**) Frequency of immobility episodes in the FST, Control = 17, CGS = 17. (**b**) Total ambulation in OFT, Control = 14, CGS = 19. (**c**) Number of entries into open arms in EZM, Control = 17, CGS = 19. Data presented as mean + SEM. Unpaired 2-tailed *t* test for FST or mixed linear factorial ANOVA for OFT where interval was a repeated measures factor.

1. Chourbaji S, et al. Differences in mouse maternal care behavior - is there a genetic impact of the glucocorticoid receptor? *PloS One*. 2011; **6**: 19218-19224.

2. Pawluski JL, Galea LAM. Reproductive experience alters hippocampal neurogenesis during the postpartum period in the dam. *Neuroscience.* 2007; **149**: 53–67.

3. Molet J, et al. Fragmentation and high entropy of neonatal experience predict adolescent emotional outcome. *Transl Psychiatry*. 2016; **6**: e702.

4. Davis EP, et al. Exposure to unpredictable maternal sensory signals influences cognitive development across species. *Proc Natl Acad Sci USA*. 2017; **39**: 10390-10395.

5. Baram TZ, et al. Fragmentation and unpredictability of early-life experience in mental disorders. *Am J Psychiatry*. 2012; **9**: 907-915.

6. Gerecsei LI, et al. Gestational exposure to the synthetic cathinone methylenedioxypyrovalerone results in reduced maternal care and behavioral alterations in mouse pups. *Front Neurosci*. 2018; **12**: 27-41.

7. Hess SE, et al. Home Improvement: C57BL/6J Mice Given More Naturalistic Nesting Materials Build Better Nests. *J Am Assoc Lab Anim Sci JAALAS*. 2008; **47**: 25–31.

8. Deacon RMJ. Assessing nest building in mice. *Nat Protoc*. 2006; **1**: 1117–1119.

9. Boyle MP, et al. Acquired deficit of forebrain glucocorticoid receptor produces depression-like changes in adrenal axis regulation and behavior*. Proc Natl Acad Sci U S A*. 2005; **102**: 473–478.

10. Kuerbitz J, et al. Loss of Intercalated Cells (ITCs) in the Mouse Amygdala of Tshz1 Mutants Correlates with Fear, Depression, and Social Interaction Phenotypes. *J Neurosci*. 2018; **38**:1160–1177.

11. Amos-Kroohs RM, et al. Neurobehavioral phenotype of C57BL/6J mice prenatally and neonatally exposed to cigarette smoke. *Neurotoxicol Teratol*. 2013; **35**: 34–45.

12. Carlin JL, Grissom N, Ying Z, Gomez-Pinilla F, Reyes TM. Voluntary exercise blocks western diet-induced gene expression of the chemokines CXCL10 and CCL2 in the prefrontal cortex. *Brain Behav Immun*. 2016; **58**: 82-90.

13. Chen A, Huang BS, Wang HW, Ahmad M, Leenen FH. Knockdown of mineralocorticoid or angiotensin II type 1 receptor gene expression in the paraventricular nucleus prevents angiotensin II hypertension in rats. *J Physiol*. 2014; **592**: 3523–3536.

14.  Charan J, Kantharia ND. How to calculate sample size in animal studies? *J. Pharmacol. Pharmacother*. 2013; **4**: 303–306.
