# Supplementary material for: Chronic psychosocial stress during pregnancy affects maternal behavior and neuroendocrine function and modulates hypothalamic CRH and nuclear steroid receptor expression
Source: Transl Psychiatry. 2020 Jan 16;10:6. doi: 10.1038/s41398-020-0704-2 (PMC7026416; doi:10.1038/s41398-020-0704-2)
Supplement: Supplementary file 2 — Supplementary Figure Table S1 [file 41398_2020_704_MOESM2_ESM.docx]

| Table S1 |  |  |  |
| --- | --- | --- | --- |
| Pregnancy and litter characteristics |  |  | |
|  | Control (N=17) | CGS (N=17) | |
| Length of pregnancy | G19.5 | G19.5 | |
| Dystocia events (total n per group) | 2 | 3 | |
| Dead pups (total n per group) | 2 | 2 | |
